# The effect and safety of Bushen Huoxue Method combined with cyclophosphamide in the treatment of systemic lupus erythematosus: A protocol for systematic review and meta-analysis

**DOI:** 10.1097/MD.0000000000031453

**Published:** 2022-11-25

**Authors:** Huihui Mao, Fengming Dai, Zonghua Du

**Affiliations:** a Department of Nephropathy and rheumatology, The central hospital of enshi tujia and Miao autonomous prefecture, Enshi, Hubei Province, China

**Keywords:** Bushen Huoxue Method, cyclophosphamide, meta-analysis, protocol, systemic lupus erythematosus

## Abstract

Systemic lupus erythematosus (SLE) is an autoimmune disease that involves multiple systemic organs. Bushen Huoxue Method (BSHXM) is a traditional Chinese medicine treatment, which is used for the treatment of SLE combining with cyclophosphamide. However, no systematic review has been performed to describe its effectiveness. This study provides a protocol for systematic review and meta-analysis of currently published randomized controlled trials (RCTs) reporting the combination of BSHXM and cyclophosphamide for the treatment of SLE, thus providing evidences to support clinical practice.

**Methods:**

RCTs reporting the combination of BSHXM and cyclophosphamide for the treatment of SLE before October 2022 will be searched in the online databases, including the PubMed, Cochrane, Embase, Web of Science, CNKI, Wanfang, VIP databases and CBM. The Cochrane Risk of Bias 2 (RoB2) tool will be used to evaluate the quality of included RCTs. Meta-analysis will be performed using Stata 14.0.

**Results:**

Results to be published in a peer-reviewed journal providing evidence for the efficacy and safety of the combination of BSHXM and cyclophosphamide on the treatment of SLE.

**Conclusions:**

This study will provide a strong basis for the effectiveness and safety of the combination of BSHXM and cyclophosphamide on the treatment of SLE.

## 1. Introduction

Systemic lupus erythematosus (SLE) is autoimmune-mediated and diffuse connective tissue disease that is highlighted by an immune inflammatory response.^[1]^ The clinical manifestations of SLE are complex, with fever and facial butterfly erythema as the typical manifestations. SLE not only damages the skin, mucous membranes and joints, but also often harms internal organs like the heart, lung, liver, kidney, and blood system. It eventually causes multisystem damage.^[2]^ Conventional Western medicine for the treatment of SLE includes glucocorticoids, immunosuppressants, nonsteroidal anti-inflammatory drugs, and biological agents.^[3,4]^ However, long-term use of drugs causes adverse events, which should not be underestimated.^[5]^ Therefore, novel treatment methods need to be developed.

SLE belongs to the category of “butterfly sore,” “horse sore,” and “sun sore” in the traditional Chinese medicine (TCM).^[6]^ TCM has good therapeutic prospects and achieves good therapeutic outcomes for SLE due to its low side effects and multi-targeting characteristics.^[7]^ The TCM theory believes that SLE is usually caused by congenital deficiency of endowment, deficiency of kidney yin, imbalance of yin and yang, imbalance of qi and blood, and stagnation of qi and blood stasis.^[8]^ Deficiency of kidney yin, combined with external evil from six sexes, or exertion or emotional injury, resulting in deficiency of true yin, internal heat stasis, paralysis and obstruction of veins and channels, external invasion of the skin, and internal organ damages.^[9]^ Tonifying the kidneys is the general principle for the treatment of SLE. The long-term course of SLE would result in the entrance of evil into the ligaments and blockage caused by the stasis of blood.^[10]^ Bushen Huoxue Method (BSHXM) is a common treatment method for SLE in clinical practice, which has achieved good therapeutic effects after long-term clinical practice.^[11,12]^

In China, BSHXM is often used in the combination with cyclophosphamide for the treatment of SLE. However, systematic evaluation on its effects is scant. In this study, we will collect clinical evidence of the combination of BSHXM and cyclophosphamide for the treatment of SLE, thus assessing its efficacy and safety by systematic evaluation and meta-analysis.

## 2. Methods

### 2.1. Study registration

This protocol has been registered on the International Prospective Register of Systematic Reviews (PROSPERO) with registration number CRD42022357993, basing on the Preferred Reporting Items for Systematic Reviews and Meta-analysis Protocols (PRISMA-P) statement guidelines.^[13]^

### 2.2. Inclusion criteria for study selection

#### 2.2.1. Types of studies.

RCTs reporting the combination of BSHXM and cyclophosphamide for the treatment of SLE.

#### 2.2.2. Types of participants.

SLE is diagnosed based on the guidelines proposed by the American College of Rheumatology in 1997.^[14]^ All patients are clearly diagnosed with SLE, with no restrictions on gender, age, or case origin.

#### 2.2.3. Types of interventions.

The combination BSHXM and cyclophosphamide is given to SLE patients of intervention group, and cyclophosphamide alone is given to those of control group. Conventional treatment and care are given to both groups.

#### 2.2.4. Outcomes

1. Primary outcome: Total effective rate.
2. Secondary outcomes: Blood sedimentation, IgA, IgG, C3, C4, and the incidence of adverse events.

### 2.3. Exclusion criteria

1. Duplicate or unpublished literatures.
2. Non-RCTs.
3. Literatures with unclear diagnostic criteria.
4. Literature review or case report.

### 2.4. Searching strategy

RCTs reporting the combination of BSHXM and cyclophosphamide for the treatment of SLE before October 2022 will be searched in the online databases, including the PubMed, Cochrane, Embase, Web of Science, CNKI, Wanfang, VIP databases, and CBM. MeSH terms and free terms will be searched. The searching strategy in the PubMed was shown in Table 1.

**Table 1:** Search strategy in PubMed database.

Number	Search terms
#1	Lupus Erythematosus, Systemic[MeSH]
#2	Libman-Sacks Disease[Title/Abstract]
#3	Lupus Erythematosus Disseminatus[Title/Abstract]
#4	Systemic Lupus Erythematosus[Title/Abstract]
#5	Disease, Libman-Sacks[Title/Abstract]
#6	Libman Sacks Disease[Title/Abstract]
#7	or/1-6
#8	Bushen Huoxue [Title/Abstract]
#9	Randomized Controlled Trials as Topic[MeSH]
#10	Clinical Trials, Randomized[Title/Abstract]
#11	Controlled Clinical Trials, Randomized[Title/Abstract]
#12	Trials, Randomized Clinical[Title/Abstract]
#13	Random*[Title/Abstract]
#14	or/9-13
#15	#7 and #8 and #14

### 2.5. Data collection and analysis

#### 2.5.1. Literature screening and data extraction.

**Figure 1.**
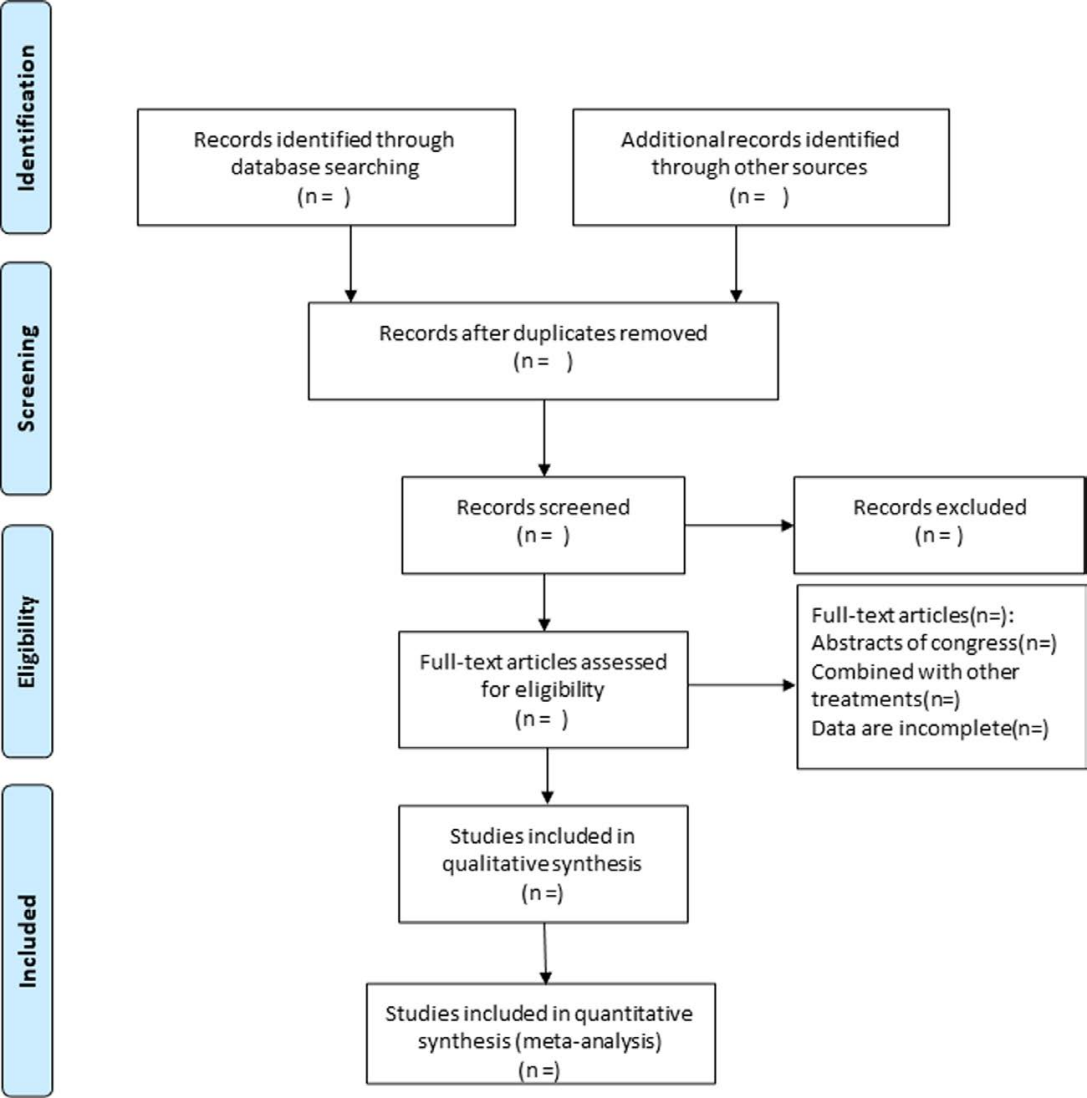
Flow diagram of study selection process.

All literatures will be independently screened by 2 researchers. After reviewing the titles or abstracts, qualified literatures will be further reviewed for the full-text according to PICOS principles and inclusion and exclusion criteria. Any inconsistency will be discussed by a third investigator. The following information will be extracted: authors, year of study inclusion, sample size, number of participants and dropout rates, type and content of intervention, dose, and duration of intervention. The screening flow chart of this study was presented in Figure 1.

#### 2.5.2. Assessment of the risk of bias.

The Risk Bias Assessment Tool for Randomized Controlled Trials Revision 2. 0 (RoB2) will be used to assess risk bias in the included literature.^[15]^ RoB2 contains five modules, namely, bias arising from the randomization process, bias from deviations from the established intervention, bias from missing outcome data, bias from outcome measures, and bias from selective reporting of outcomes.

#### 2.5.3. Measures of therapeutic efficacy.

Relative risk and weighted mean difference will be calculated for dichotomous variables and continuous variables, respectively. Corresponding 95% confidence interval will be calculated.

#### 2.5.4. Management of missing data.

Missing data in the included literatures will be acquired by e-mailing the corresponding author.

#### 2.5.5. Assessment of heterogeneity and data synthesis.

Stata 14. 0 software will be used for data analysis. The heterogeneity will be examined by *Q* test and measuring *I*^2^ value. *P* > .1 and *I*^2^ < 50% suggests a low heterogeneity, and the fixed-effects model will be used; Otherwise, the random-effects model will be used.

#### 2.5.6. Assessment of publication biases.

Funnel plots will be made to qualitatively assess the publication bias of the included literatures.^[16–18]^

#### 2.5.7. Subgroup analysis.

Subgroup analyses based on the patient age, race, BSHXM, and disease subtype will be performed to explore potential sources of heterogeneity.

#### 2.5.8. Sensitivity analysis.

Sensitivity analysis will be performed by eliminating one literature at one time and calculating the remaining data. The results of the meta-analysis will be considered robust and reliable if there is no significant change in the data of remaining literatures.

#### 2.5.9. Ethics and dissemination.

As this study is a protocol for systematic review and meta-analysis, it does not involve individual patient data and therefore does not require ethical approval. The results of this study will be published in a peer-reviewed journal.

## 3. Discussion

SLE is an autoimmune disease that involves multiple systemic organs. Due to SLE-induced humoral immune dysfunction, glucocorticoids and immunosuppressants are used to the treatment. However, their long-term use produces significant adverse events..^[19,20]^ The combination of BSHXM and cyclophosphamide for the treatment of SLE has the unique advantage in improving clinical efficacy and reducing adverse effects. However, relevant RCTs reporting the combination BSHXM and cyclophosphamide for the treatment of SLE vary a lot, and their efficacy and safety are unclear. This systematic review will provide a comprehensive evaluation of the efficacy and safety of the combination of BSHXM and cyclophosphamide for the treatment of SLE. Evidence from this systematic review may be beneficial to SLE patients and clinicians applying BSHXM.

## Author contributions

Resources: Fengming Dai.

Data collection: Fengming Dai.

Writing—original draft: Huihui Mao and Zonghua Du.

Writing—review and editing: Huihui Mao and Zonghua Du.

Funding support: Zonghua Du.

Supervision: Zonghua Du.

Software operating: Fengming Dai.

**Conceptualization:** Huihui Mao, Zonghua Du.

**Data curation:** Huihui Mao, Fengming Dai.

**Funding acquisition:** Zonghua Du.

**Investigation:** Huihui Mao, Fengming Dai.

**Methodology:** Fengming Dai.

**Resources:** Fengming Dai, Zonghua Du.

**Software:** Fengming Dai.

**Software:** Zonghua Du.

**Validation:** Zonghua Du.

**Visualization:** Zonghua Du.

**Writing—original draft:** Huihui Mao, Zonghua Du.

**Writing—review and editing:** Huihui Mao.
